# Privacy Preserving Quantum Anonymous Transmission via Entanglement Relay

**DOI:** 10.1038/srep26762

**Published:** 2016-06-01

**Authors:** Wei Yang, Liusheng Huang, Fang Song

**Affiliations:** 1 School of Computer Science and Technology, University of Science and Technology of China, Hefei, 230026, China; 2 Suzhou Institute for Advanced Study, University of Science and Technology of China, Suzhou, 215123, China; 3 Institute for Quantum Computing, University of Waterloo, 200 University Ave. West, Waterloo, Ontario, Canada.

## Abstract

Anonymous transmission is an interesting and crucial issue in computer communication area, which plays a supplementary role to data privacy. In this paper, we put forward a privacy preserving quantum anonymous transmission protocol based on entanglement relay, which constructs anonymous entanglement from EPR pairs instead of multi-particle entangled state, e.g. GHZ state. Our protocol achieves both sender anonymity and receiver anonymity against an active adversary and tolerates any number of corrupt participants. Meanwhile, our protocol obtains an improvement in efficiency compared to quantum schemes in previous literature.

One functionality of computer communication is under construction to ensure secure information transfer among participants^1^,^2^. So far, most applications in computer communications focus on the secrecy of messages, which means no one except sender and receiver learns the content of the messages. However, identities of sender and receiver can also be sensitive information in many situations, such as privacy-preserving identity management^3^,^4^, electronic auction^5^, signature-based scheme^6^, electronic voting^7^, and anonymous email^8^, etc. Therefore, protecting anonymity, the secrecy of identity, has stepped into the spotlight of scholars. Meanwhile, the property of anonymity serves as a valuable building block in constructing other protocols. Examples are Byzantine agreement^9^, key distribution^10^ and digital pseudo signature^11^.

In the classical world, there exist generally three types of approaches towards anonymity. The first type employs a trusted third party^12^,^13^, which forwards messages while hiding the identity of the original sender. The second type uses a chain of servers to randomize the ordering of messages. The most popular instance is Chaum’s MixNets^8^. The last type achieves unconditional (information-theoretical) security. Chaum first introduced one such protocol, known as *Dining Cryptographers Problem* in 1988^14^. The scheme allows anonymous broadcast of classical messages. Meanwhile, different forms of anonymous channels can be constructed based on the technique presented in ref. 8, all of which are denoted as DC-nets. An example is anonymous broadcast, message transfer with anonymous sender and anonymous receiver.

In the quantum world, the first quantum anonymous transmission (QAT) protocol was proposed in ref. 15, which allows anonymous communication of classical information by virtue of quantum mechanics. After that, Christandl and Wehner gave explicit definitions and models for the anonymous transfer of classical information and quantum information in 2005^16^. A key notion proposed by them is *anonymous entanglement*, with which two parties can perform quantum teleportation protocols^17^ so as to achieve the goal of anonymous transfer of quantum information. Anonymous entanglement is also the key ingredient of our scheme to be presented in this paper, but realized via a straightforward and efficient way. However, an assumption in ref. 18 is the *n* participants share the trusted 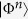 beforehand, which is impossible with dishonest participants. Ref. 18 overcomes this drawback by compromising the anonymity. After ref. 18 , Brassard *et al*. proposed an information-theoretically secure protocol for the anonymous transfer of quantum information^19^. Their protocol also protects the privacy of the quantum message perfectly. Nonetheless, they built the protocol heavily based on several sub-protocols in ref. 20. These protocols have to terminate in presence of even one corrupt participant. This renders the scheme in ref. 19 prone to abort. In addition, all three protocols^16^,^18^,^19^ establish anonymous entanglement by employing multi-particle entanglement, which consumes much quantum resource. Here lies one of our motivations: to establish anonymous entanglement via less resource.

In fact, some work has been conducted to establish anonymous entanglement using less resource based on single photons. In 2010, Wang *et al*. presented an excellent QAT scheme (WWZ10)^21^. They employ single photons to construct anonymous entanglement instead of multi-partite entangled states in their protocol. The WWZ10 scheme shares advantages of low photons costs and low communication complexity, and thus be “economical”. Their solution requires only *O*(3) qubits to construct an anonymous entangled state, which is very economical and efficient. However, the scheme is vulnerable against collusion attack. For example, if participant 1 and participant 3 collude with each other and one of them is notified to be the receiver, then they will get the identity of participant 2 with a certain probability. Another QAT scheme using single photons was proposed by Ronghua Shi *et al*.^22^. They demonstrated an anonymous quantum communication (ACQ) via the nonmaximally entanglement state^23^ based on the dining cryptographer problem. However, their protocol also has security loophole. Using the attacking method introduced in ref. 24, half of the secret bits of the sender in ref. 22 will be disclosed. This may suggest that single photons are not desirable resources to establish anonymous entanglement.

In this paper, we present a privacy preserving anonymous transmission protocol for quantum messages. In our protocol, we utilize EPR pairs to generate anonymous entanglement rather than using multi-particle entangled state. Our protocol achieves both sender anonymity and receiver anonymity against an active adversary and tolerates any number of corrupt participants. Thorough analysis and comparisons with other QAT protocols manifest that our protocol outperforms previous schemes in efficiency and conciseness.

## Preliminaries

We consider the same scenario as in refs 16,19. Within a set of *n* participants that are consecutively numbered, the sender intends to transmit a private quantum message to the receiver while protecting the anonymity of both sender and receiver. For the sender, anonymity means that he is unknown to all other participants, i.e. even the receiver cannot get the identity of the sender; for the receiver, it means no one except the sender knows his identity. This setting can be regarded as an instance of Secure Multi-party Computation (SMC), so we review two mostly considered security models in SMC^25^: an adversary controls and corrupts a portion of participants in either a passive or an active manner. In the passive model (also called semi-honest model or honest-but-curious model), corrupt participants follow the protocol honestly, but collude with each other by gathering all the information and then sharing them in order to get more information than their common inputs and outputs. In the active model (also called malicious model), corrupt participants may take active steps to disrupt the execution of the protocol. In our paper, we justify our protocol in the case of active adversary and assume that the set of corrupt participants is fixed before the protocol starts (defined as non-adaptive).

We herein introduce two tools that are useful in the construction of our protocol. They are anonymous broadcast of classical message^14^ and notification protocol^20^.

**Theorem 1.** (*Anonymous broadcast of classical message*^20^) There are *n* participants within which one sender has a message *msg* to broadcast. There exists one anonymous broadcast protocol so that: (1) Everyone receives *msg*. (2) An adversary controlling *t* participants can correctly guess the identity of the sender with probability no larger than 1/(*n *− *t*). (3) Any disruption of the protocol will be detected.

**Theorem 2.** (*Notification protocol*^20^) There exists a notification protocol in which any player can notify other players of his choice. Each player’s output is one private bit specifying if he has been notified at least once; this value is correctly computed with probability exponentially close to 1.

In Theorem 1 and Theorem 2, classical broadcast channels are needed. According to ref. 20, there are generally two kinds of broadcast channels. The first one is the regular broadcast channel. It is an authentic broadcast channel for which the sender is sure that all participants obtain the same message and they are aware of who is the sender. The second kind is called the simultaneous broadcast channel. This a collection of broadcast channels which can prevent one participant from inputting rely on any other participant’s input. In the context of the present paper, we use *broadcast channel* to denote a regular classical broadcast channel.

## Design of the QAT Protocol via Entanglement Relay

In our protocol, we make the same assumptions as those in ref. 19: a classical broadcast channel as well as a private authenticated quantum channel is between each pair of participants.

### Building Blocks

**Figure 1:**
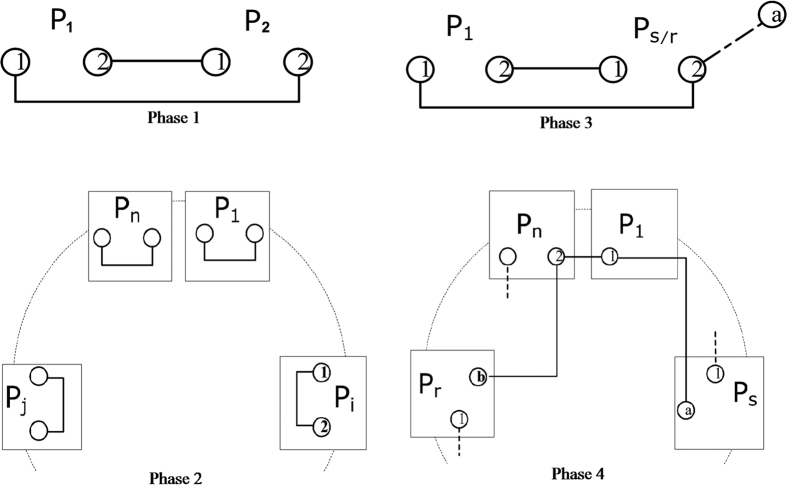
Different phases during the relay. Phase 1: beginning. Phase 2: entanglement swapping. Phase 3: sender/receiver C-NOT. Phase 4: ending state.

Our way of generating anonymous entanglement works like a *relay*. We suppose each participant holds one pair of EPR at the beginning of the protocol (without loss of generality, we suppose all of the EPR pairs are in state 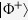), as shown in phase 1 of Fig. 1. We use 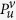 to indicate the related qubit, where *v* = 1 or 2 is the first or second qubit of the EPR pair held by the *u*th participant. Now the relay starts.

A randomly chosen participant (here we designate the chosen one as *P*_1_) transmits his second qubit (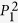) to his right-hand neighbor, who then performs a Bell-State Measurement (BSM) on 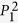 and the first one of his EPR pair 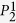. This will result in entanglement swapping, see phase 2), between the two bell states held by *P*_1_ and *P*_2_. Similarly *P*_2_ transmits 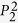 to the next one, and this relay continues until 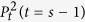 reaches the sender *P*_*s*_ (the receiver *P*_*r*_’s behavior is identical to that of sender *P*_*s*_). *P*_*s*_ not only performs a BSM on 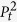 and 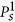, but after BSM he performs a C-NOT transformation on 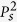 and an additional qubit *Q*^*a*^ in state 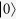, where 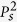 acts as the control qubit and 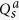 acts as the target qubit (phase 3 of Fig. 1).

It is clear that at the end of the relay, as shown in phase 4 of Fig. 1, the four separate qubits held by *P*_1_, *P*_*s*_, *P*_*r*_ and *P*_*n*_ stay in the state 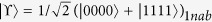 where *b* denotes the additional qubit introduced by the receiver. *P*_1_ and *P*_*n*_ then run the last step by measuring 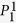 and 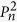 in Hadamard basis 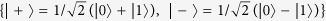 respectively. This will cast *Q*^*a*^ and *Q*^*b*^ into 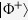 (two measurement outcomes are identical) or 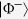 (two measurement outcomes differ, and 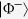 can be transformed into 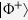 easily, see below), as shown in Eq. (1). Thus after this round of relay, we have successfully built one instance of anonymous entanglement 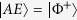 between *P*_*s*_ and *P*_*r*_.


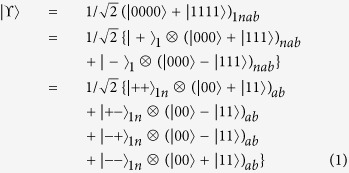


Note that in the description above, different possible outcomes of BSM (entanglement swapping) may occur in each step of the relay. In order for the sender *P*_*s*_ to transform the final Bell state to the desired 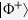, we require that each participant anonymously broadcasts the outcome of his BSM. Then *P*_*s*_ knows the final entangled state 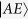, and corresponding unitary transformations can be performed on his qubit of 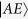 to obtain 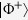: 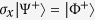, 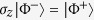, and 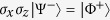, where *σ*_*x*_, *σ*_*z*_ are Pauli operators. Protocol 1 gives a concise summary.

**Protocol 1.** *Establishment of Anonymous Entanglement*.

**Goal:** anonymously sharing 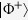 between sender and receiver in a group of *n* members.

**Requirements:** each participant hold one EPR pair in 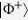, a classical broadcast channel, C-NOT gate.

1. *n* participants are ordered by *P*_1_, *P*_2_, 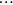, *P*_*n*_. One participant is chosen randomly (assume to be *P*_1_) to initiate the protocol by sending his second qubit of 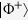 to his right-hand neighbor.

2.Each participant performs the BSM in turn to realize entanglement swapping and continue the relay till the *n*th participant’s operation. During this procedure, the sender *P*_*s*_ (the same for receiver *P*_*r*_) introduces an additional qubit *Q* in 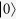, and performs C-NOT on his second qubit (control qubit) and *Q* (target qubit) after his BSM.

3. Every participant (except *P*_1_) anonymously broadcasts the outcome of his BSM. *P*_*s*_ performs corresponding unitary transformations on *Q*.

4. *P*_1_ and *P*_*n*_ perform measurements in Hadamard basis, and broadcast the outcomes. If the two values differ, *P*_*s*_ performs *σ*_*z*_ on *Q* otherwise he does nothing and the protocol completes.

In practice, to prevent malicious behavior by adversary and corrupt participants, sender and receiver have to employ additional methods to protect their anonymity and data privacy. See protocol 2.

**Protocol 2.** *Malicious Act Detection*.

**Goal:** Detecting malicious act with probability exponentially close to 1.

**Requirements:** anonymous broadcast channel for classical messages.

For (*α* + *β*) instances of 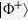 that need verifying:

1. *P*_*s*_ and *P*_*r*_ measure *α* pairs of *Q*^*a*^ and *Q*^*b*^ in Hadamard basis.

2. *P*_*s*_ and *P*_*r*_ measure *β* pairs of *Q*^*a*^ and *Q*^*b*^ in computational basis.

3. *P*_*s*_ and *P*_*r*_ publish the outcomes using the anonymous broadcast channel.

4. If different outcomes appear, then malicious acts have occurred, and the protocol aborts.

We can see the probability that a malicious act passes protocol 2 without being detected is at most 1/2^[*α,β*]^, where [*α*, *β*] means the smaller one of *α* and *β*. Actually, any type of deviation from 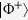 can be detected effectively by Protocol 2, which will be explained in detail in next section.

### Protocol for QAT

Up to now, we have discussed all necessities of constructing a full protocol. We now present it in Protocol 3.

**Protocol 3.** *Anonymous Transmission of Quantum Message*.

**Goal:** Transmitting a message of *m*-qubit from an anonymous sender to an anonymous receiver, protecting the privacy of the message.

**Requirements:** requirements in Protocols 1 and 2, Notification Protocol.

1. The sender *P*_*s*_ notifies the receiver *P*_*r*_ via the Notification Protocol.

2. Execute Protocol 1 for 2(*m *+ *k*) times to share 2(*m *+ *k*) instances of 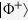 between *P*_*s*_ and *P*_*r*_ anonymously.

3. For these instances, execute Protocol 2. If the detection passes, the protocol continues; otherwise the protocol aborts and restarts. The protocol will be terminated if the number of abortions reaches a larger enough predetermined parameter.

4. *P*_*s*_ transmits the quantum message through teleportation using the *m* instances of 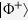, and then anonymously broadcasts the teleportation bits.

5. *P*_*r*_ reconstructs the quantum message. Then he anonymously broadcasts one bit to indicate whether or not the reconstruction has succeeded. If true, the protocol terminates successfully.

6. *P*_*r*_ teleports the quantum state resulting from step 5 back to *P*_*s*_ using the remaining *m* instances of 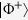. Then he broadcasts the teleportation bits anonymously.

7. *P*_*s*_ reconstructs the quantum message. The protocol completes.

## Analysis and Proof

### Security

From previous discussions, we can see that our protocol preserves the anonymity of sender and receiver while the privacy of the quantum message is also protected. Formally, we have the fowling conclusion:

**Theorem 3.** (*Security*) Protocol 3 tolerates any number of corrupt participants, no matter whether they are controlled by a passive or an active adversary. The anonymity of sender and receiver is perfectly protected. The privacy of the message is secure except with a negligible probability.

#### Proof

Obviously, if all the participants are honest, 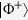 is faithfully and anonymously shared between sender and receiver, because apparently there are no detectable differences in the behaviors of the anonymous sender *P*_*s*_ and receiver *P*_*r*_ with those of the other participants. This is true even in presence of passive adversary, since any number of honest-but-curious participants can never reveal the identity of *P*_*s*_ and *P*_*r*_, based on all information they get (BSM outcomes, Hadamard measurement outcomes, etc.) during protocol 1.

To accomplish the security proof, we will construct corresponding *simulator* for each participant who attempts to deduce the identity of sender or receiver. The general idea underlying the method of simulator is that a if a simulator for a player can emulate the execution of a protocol with only the input this player’s input and the output of the final outcome, then we can safely conclude that this protocol is secure against this player and he is not able to obtain more information about the other players’ private data. This is because the simulator itself has no knowledge about those private data. For formal definitions of simulator, view and computational indistinguishable, we refer readers to ref. 26.

Let us start with the sender anonymity. We need to present a simulator for each party (except the sender) view. The simulator for participant *i* (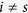) is denoted as *S*_*i*_. On input 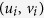, where *u*_*i*_ is the local input to participant *i* other than the sender, and *v*_*i*_ is his local output, *S*_*i*_ selects uniformly and randomly a Bell state *t*_*i*_ from the set 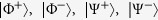 and output 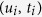. We now show that this output is distributed identically to the view of participant *i*. Note that the BSM outcome for participant *i* is totally random and its value is taken from one of the four states 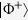, 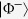, 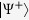, and 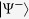 uniformly, therefore there is no method to distinguish from *v*_*i*_ and *t*_*i*_ (formally, we say that they are computational indistinguishable). According to the basic idea of simulator, we are convinced that the sender anonymity is protected in our scheme. Similarly, we can construct simulators to prove receiver anonymity. We omit it here for brevity.

Till now, we know that Protocol 3 achieves both sender and receiver anonymity, and thus Theorem 3 holds.

In the following, we give some typical attacking strategies that an adversary may adopt to demonstrate the correctness of Protocol 3.

A direct means of attack for a malicious participant *P*_*m*_ is to introduce also an additional qubit and perform C-NOT transformation to build a correlation with the anonymous entangled state 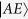 shared by *P*_*s*_ and *P*_*s*_. Thus the final quantum system will be in the state 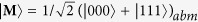, where *m* indicates the additional qubit *Q*^*m*^ introduced by *P*_*m*_. *P*_*m*_ thus may reveal the identity of *P*_*s*_ or *P*_*r*_. If *P*_*s*_ later uses this state to transmit quantum messages via teleportation, we know that either *P*_*m*_ or *P*_*r*_ can reconstruct the initial message, which destroys the privacy of the message. In order to prevent this, we observe a fact from a simple observation, that in case 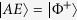, if *P*_*s*_ and *P*_*s*_ measure *Q*^*a*^ and *Q*^*b*^ in Hadamard basis respectively, they always have the same outcomes because


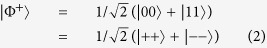


However, in case *Q*^*m*^ is introduced, we see that:


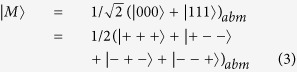


Therefore, with a chance of 1/2, *P*_*s*_ and *P*_*r*_ will obtain different outcomes. If the participants run Protocol 1 for a sufficiently large number of rounds to generate a number of 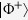 (possibly 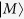) between *P*_*s*_ and *P*_*r*_, they can then select a portion of them (e.g. *k* pairs) and perform measurement in Hadamard basis. After comparison (using anonymous broadcast) of the outcomes, they have a high probability (1 − 1/2^*k*^) of detecting the malicious behavior. We would like to emphasize that no matter how many malicious participants apply this strategy, they would never succeed, that is because measurements of *Q*^*a*^ and *Q*^*b*^ are independent of the rest qubits. Thus the increase of entangled particles makes no difference as the malicious action will be caught with probability 1/2, and enough rounds of detection will improve the probability exponentially close to 1.

**Figure 2:**
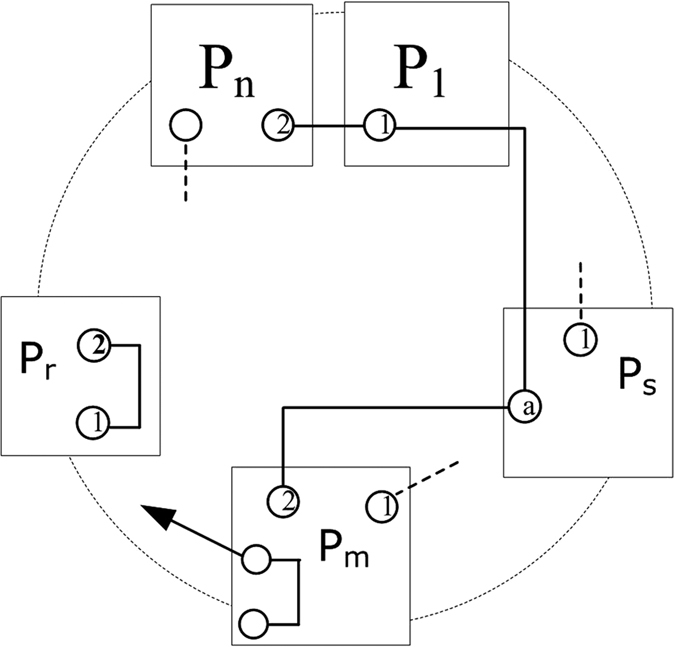
Replacing entanglement by a malicious participant. keeps and sends to the neighbor.

**Figure 3:**
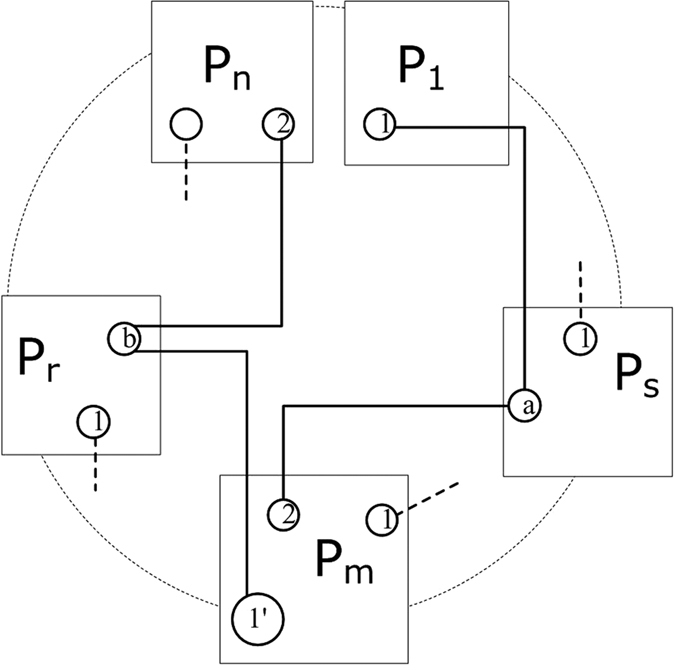
At the end of the relay.

Another trick a malicious participant *P*_*m*_ may play is to replace 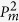 with one qubit of an EPR pair that is prepared by himself (Figs 2 and 3). Here we assume that *P*_*m*_ is between *P*_*s*_ and *P*_*r*_, because both the cases that *P*_*m*_ is before *P*_*s*_ and *P*_*m*_ is after *P*_*r*_ would fall into the above discussion.

After the relay, there exist two instances of entanglement: one held by *P*_1_, *P*_*s*_ and *P*_*m*_ in state 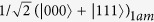; the other by *P*_*m*_, *P*_*r*_ and *P*_*n*_ in state 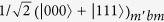. Now, the qubits possessed by *P*_*s*_ and *P*_*r*_ are unrelated. However, if they measure in Hadamard basis as well, so long as inconsistency (different measurement outcomes) happens, they should know that malicious participants exist. Moreover, the probability of the inconsistency happening is also 1/2, which will render the detection probability exponentially close to 1 with sufficient trials.

Here we again place no limitation on the number of corrupt participants. This is because the final quantum system will always be in the two states shown above. The only difference is which participants get to keep them, besides *P*_s_ and *P*_*r*_. Thus the same detection applies naturally.

Previous discussions suffice in a standard SMC model. However, some participants are just so *naughty* that they broadcast false outcomes of their measurements to fool *P*_*s*_ into performing unnecessary unitary transformation, causing 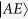 to differ from 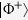. For example, suppose *P*_*S*_, *P*_*r*_, *P*_*s−*1_ and *P*_*r−*1_ all get 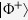 in BSM, which means 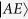 will also be in 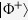 after *P*_1_’s and *P*_*n*_’s operations. However, *P*_*r*−1_ broadcasts that he obtains 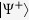, which misleads sender and receiver into thinking that 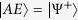. The sender will perform a *σ*_*x*_ on his qubit that turns the genuine 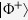 to 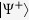. Note that 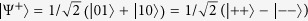 Therefore measurements in Hadamard basis will always lead to identical outcomes, which renders Protocol 2 futile. The solution lies in the fact that only 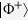 results in identical measurement outcomes in both Hadamard basis and computational basis.

Hence, we can choose two subgroups of anonymous entangled states, then measure one group in Hadamard basis and the other in computational basis. As long as differences occur, malicious behavior is detected. Therefore, protocol 2 can detect inconsistency with 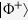, and the probability of success is exponentially close to 1. Thus any cheating strategy adopted by the adversary and the corrupt participants will be detected, which ensures the anonymity of sender and receiver. The quantum message remains private, and we teleport the message back to the sender when the receiver does not succeed in reconstructing it. This guarantees the state to be transmitted would never be destroyed even if the protocol aborts. Thus the privacy of the transmitted quantum message is also perfectly protected.

At last, let us consider an attack strategy by two corrupted participants. Let *P*_*i*_, *P*_*j*_ and *P*_*k*_ be any three consecutive participants. Provided *P*_*i*_ and *P*_*k*_ are corrupted, they collude with each other by performing as follows: *P*_*i*_ creates an EPR pair and sends one subsystem to *P*_*j*_. *P*_*j*_ does entanglement swapping (and applies the C-NOT provided he is the sender or receiver). *P*_*k*_ broadcasts the measurement outcome and forwards the other particle to *P*_*k*_. Now, provided *P*_*j*_ is not sender or receiver, *P*_*i*_ and *P*_*k*_ will share an EPR pair (thanks to the result of the *P*_*j*_'s BSM they know which one). In case *P*_*j*_ is sender or receiver, they will share a GHZ state (with *P*_*j*_), again fully specified. Now it only suffices to repeat this a number of times and discriminate between EPR and GHZ state, what is possible using certain entanglement testing. The two corrupted participants hope to verify whether *P*_*j*_ is a normal participant, or sender or receiver, via this strategy.

However, this strategy will not work either. Note in the absence of this kind of attack, *P*_*s*_ and *P*_*r*_ will share a 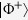 in a round of relay. In contrast the introduction of new EPR pair and annunciation of “fake” BSM outcomes by *P*_*i*_ and *P*_*k*_ will result in *Q*^*a*^ and *Q*^*b*^ being uncorrelated or in a state other than 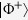. Like before, the malicious action will be detected by a number of verifications between *P*_*s*_ and *P*_*r*_. They will abort the current round of relay and restart the protocol. And, just as described in Protocol 3, the scheme will be terminated if the number of abortions reaches a predetermined parameter, which indicates too many malicious actions exist in the protocol. Moreover, consider the extreme situation where the number of corrupted participants reach *n *− 2 (except sender and receiver). Then they have no better method to distinguish between sender and receiver than by solely making a guess. Hence their chance of learn who is sender or receiver is not larger than 1/2.

### Efficiency and Robustness

In what follows we would like to discuss the efficiency and robustness of our scheme. In sharp contrast to previous protocols, the main quantum resources we utilize in our protocol are EPR pairs instead of generalized GHZ state. From present day techniques, multi-party entangled states are relatively difficult to realize. So far, the best work is done by W. B. Gao *et al*. whose group realized entanglement of ten photons^27^. Thus, our protocol envisages an application in the near future. Meanwhile, our protocol itself costs much fewer qubits. See the *Entanglement Verification* process in ref. 19, for example, each participant makes 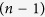 pseudo copies of his qubit. This simple operation would consume 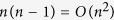 qubits. In our detection protocol, we make use of 2*k* instances of 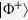, while the success probability approaches 1 exponentially with *k*. The major difference is that we only require sender and receiver to operate the detection (without compromising anonymity, of course), but in ref. 19, all the participants should be included in order to keep anonymity. Meanwhile, after one round of protocol 1, every participant (except *P*_1_ and *P*_*n*_) still keeps one EPR pair because entanglement swapping leaves 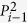 and 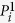 in one of the four Bell states. Thus, the total number of EPR pairs we need in Protocol 3 is just 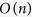. Moreover, ref. 19 takes advantage of a few sub-protocols that are complicated to run, e.g. quantum authentication. We only require anonymous broadcast and notification protocols in our proposal and this simplifies the execution of our protocol. Table 1 gives a comparison between several related protocols.

Note that ref. 19 utilizes several classical protocols proposed in ref. 20. These protocols share a common feature that a single corrupt participant can cause the protocol to abort, and this in return makes the protocol of ref. 19 prone to abort. Our protocol, however, takes advantage of a detection protocol which ensures that we terminate early in presence of malicious acts. If the detection passes, no disruption can cause the protocol to abort afterwards, except in the process of anonymous broadcast. However, we know from Theorem 2 that anyone who disrupts it will get caught and excluded in the next execution of the protocol. Thus our protocol stays more robust than ref. 19. Obviously, we also save time and the (quantum) resources used in the remaining steps of the protocol.

Nonetheless, we should pay attention to a problem that arises from step 3 in Protocol 3. As readers may have envisioned, how can *P*_*s*_ and *P*_*r*_ agree on which *k* of 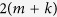 instances should be measured in Hadmard basis and which *k* instances should be measured in computational basis? If they choose completely at random, the probability that malicious participants are caught will be reduced dramatically. Moreover, in the worst case where not a single pair of choices accord, the detection protocol fails and there are only 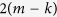 instances of 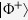 remaining. Our solution is to add one step of anonymous broadcast for *P*_s_, during which he broadcasts his choices (say, the 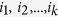
th in Hadamard basis; the 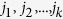
th in Computational basis). We can see this does work and makes no harm to the anonymity of the sender. Other strategies are also possible. For example, each participant shares a string of bits with everyone else in advance indicating the agreement. We will not elaborate on this issue, so long as our solution can resolve this problem effectively.

## Summary

In this paper, we have presented a privacy preserving protocol for the anonymous transmission of quantum messages, where EPR pairs are used to construct anonymous entanglement. We have shown that our protocol works more efficiently and robustly than protocols in prior literature.

So far, we have not discussed the case of multiple senders. Of course, strategies used in related literature, like collision detection^20^, can be applied to our protocol naturally. However, as mentioned in ref. 19, collision detection may reveal information on the number of honest would-be senders. Thus we wish to find effective ways to handle this in the future, probably following the line of simultaneously sharing multiple instances of anonymous entanglement between different sender-receiver pairs. This will be our future work.

## Additional Information

**How to cite this article**: Yang, W. *et al*. Privacy Preserving Quantum Anonymous Transmission via Entanglement Relay. *Sci. Rep*. **6**, 26762; doi: 10.1038/srep26762 (2016).

## Figures and Tables

**Table 1:** Comparison between four QAT protocols, where ERR and QC are abbreviated for “entanglement resource required” and “qubits consumed”, respectively.

Protocol	ERR	QC	Message type
Ref. 19	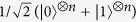	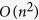	quantum
Ref. 16	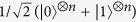	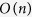	quantum/classical
Ref. 18	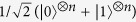	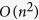	quantum
Ours	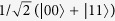	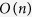	quantum
